# Supplementary material for: End Sequence Analysis Toolkit (ESAT) expands the extractable information from single-cell RNA-seq data
Source: Genome Res. 2016 Oct;26(10):1397–410. doi: 10.1101/gr.207902.116 (PMC5052061; doi:10.1101/gr.207902.116)
Supplement: Supplemental Material [file supp_gr.207902.116_Supp_Materials.tar.gz › ESAT-esat_v0.1/doc/html/classbroad_1_1core_1_1sequence_1_1_sequence-members.html]

ESAT: Member List


ESAT


- Main Page
- Related Pages
- Packages
- Classes
- Files

- Class List
- Class Index
- Class Hierarchy
- Class Members

All Classes Namespaces Files Functions Variables Enumerator Pages

- broad
- core
- sequence
- Sequence

broad.core.sequence.Sequence Member List

This is the complete list of members for broad.core.sequence.Sequence, including all inherited members.

|  |  |  |
| --- | --- | --- |
| append(String partialSequence) | broad.core.sequence.Sequence |  |
| append(char c) | broad.core.sequence.Sequence |  |
| buildKmerMap(int kmer) | broad.core.sequence.Sequence |  |
| chunk(int chunkSize, int chunkOverlap) | broad.core.sequence.Sequence |  |
| complement(String seq) | broad.core.sequence.Sequence | static |
| compute454Flow(char[] flowOrder) | broad.core.sequence.Sequence |  |
| compute454Flow(char[] flowOrder, int start, int end) | broad.core.sequence.Sequence |  |
| computeGCContent(String dnaString) | broad.core.sequence.Sequence | static |
| computeSequenceComposition(String dnaString) | broad.core.sequence.Sequence | static |
| contains(String motif) | broad.core.sequence.Sequence |  |
| countSoftMaskedBases() | broad.core.sequence.Sequence |  |
| decode(short[] shortEncodedSequence) | broad.core.sequence.Sequence | static |
| encodeSequence() | broad.core.sequence.Sequence |  |
| encodeSequence(StringBuilder bases) | broad.core.sequence.Sequence | static |
| encodeSequenceAsVector() | broad.core.sequence.Sequence |  |
| encodeSequenceIgnoreCase() | broad.core.sequence.Sequence |  |
| encodeSequenceIgnoreCase(boolean distinguishMissingSequence) | broad.core.sequence.Sequence |  |
| encodeSequenceIgnoreCase(StringBuilder sequence) | broad.core.sequence.Sequence | static |
| encodeSequenceIgnoreCase(StringBuilder sequence, boolean distinguishMissingSequence) | broad.core.sequence.Sequence | static |
| extractRegionBasedOnGC(float targetGC, int size, int buffer) | broad.core.sequence.Sequence |  |
| findUngappedSequenceChunks() | broad.core.sequence.Sequence |  |
| gcContent() | broad.core.sequence.Sequence |  |
| generateAll5Mers() | broad.core.sequence.Sequence | static |
| generateAllKmers(int k) | broad.core.sequence.Sequence | static |
| generateRandomSequence(int oligoSize) | broad.core.sequence.Sequence | static |
| get3Prime(String seq, int numBases) | broad.core.sequence.Sequence | static |
| getAntisense() | broad.core.sequence.Sequence |  |
| getEncodedSequence() | broad.core.sequence.Sequence |  |
| getEnd() | broad.core.sequence.Sequence |  |
| getGapsSize() | broad.core.sequence.Sequence |  |
| getId() | broad.core.sequence.Sequence |  |
| getLength() | broad.core.sequence.Sequence |  |
| getMatches(String s, int windowStart, int windowEnd, boolean includeRC) | broad.core.sequence.Sequence |  |
| getRefseqGeneName() | broad.core.sequence.Sequence |  |
| getRefseqRnaClassName() | broad.core.sequence.Sequence |  |
| getRefseqSpeciesName() | broad.core.sequence.Sequence |  |
| getRegion(int start, int end) | broad.core.sequence.Sequence |  |
| getRegion(SequenceRegion region) | broad.core.sequence.Sequence |  |
| getRegion(SequenceRegion region, boolean softmask) | broad.core.sequence.Sequence |  |
| getRegion(SequenceRegion region, boolean softmask, Map< String, IntervalTree< Alignments >> okRepeats) | broad.core.sequence.Sequence |  |
| getRegions(List<?extends SequenceRegion > regions) | broad.core.sequence.Sequence |  |
| getSequenceBases() | broad.core.sequence.Sequence |  |
| getSequenceBuilder() | broad.core.sequence.Sequence |  |
| getSlider(int windowSize, int overlap) | broad.core.sequence.Sequence |  |
| getSoftmaskedRegions() | broad.core.sequence.Sequence |  |
| getSubSequence(SequenceRegion region, int extension) | broad.core.sequence.Sequence |  |
| getSubSequence(String name, int start, int end, int extension) | broad.core.sequence.Sequence |  |
| getSubSequence(String name, int start, int end) | broad.core.sequence.Sequence |  |
| getSubsequence(Gene annot) | broad.core.sequence.Sequence |  |
| getSubsequence(Annotation annot) | broad.core.sequence.Sequence |  |
| getVectorEncodedSequence() | broad.core.sequence.Sequence |  |
| isForwardStrand() | broad.core.sequence.Sequence |  |
| isGap(int position) | broad.core.sequence.Sequence |  |
| LONG\_READ\_FLOW | broad.core.sequence.Sequence | static |
| mask(Annotation region, boolean softmask) | broad.core.sequence.Sequence |  |
| maskSoftmaskedRegions() | broad.core.sequence.Sequence |  |
| reverse() | broad.core.sequence.Sequence |  |
| reverseSequence(Sequence seq) | broad.core.sequence.Sequence | static |
| reverseSequence(String seq) | broad.core.sequence.Sequence | static |
| scramble() | broad.core.sequence.Sequence |  |
| Sequence(String id) | broad.core.sequence.Sequence |  |
| Sequence(String id, boolean isLarge) | broad.core.sequence.Sequence |  |
| Sequence(String id, int expectedSize) | broad.core.sequence.Sequence |  |
| setCapacity(int size) | broad.core.sequence.Sequence |  |
| setCharAt(int position, char newCharacter) | broad.core.sequence.Sequence |  |
| setForwardStrand(boolean isForwardStrand) | broad.core.sequence.Sequence |  |
| setId(String id) | broad.core.sequence.Sequence |  |
| setSequenceBases(String sequence) | broad.core.sequence.Sequence |  |
| SHORT\_ENCODED\_a | broad.core.sequence.Sequence | static |
| SHORT\_ENCODED\_A | broad.core.sequence.Sequence | static |
| SHORT\_ENCODED\_C | broad.core.sequence.Sequence | static |
| SHORT\_ENCODED\_c | broad.core.sequence.Sequence | static |
| SHORT\_ENCODED\_g | broad.core.sequence.Sequence | static |
| SHORT\_ENCODED\_G | broad.core.sequence.Sequence | static |
| SHORT\_ENCODED\_GAP | broad.core.sequence.Sequence | static |
| SHORT\_ENCODED\_N | broad.core.sequence.Sequence | static |
| SHORT\_ENCODED\_t | broad.core.sequence.Sequence | static |
| SHORT\_ENCODED\_T | broad.core.sequence.Sequence | static |
| SHORT\_READ\_FLOW | broad.core.sequence.Sequence | static |
| shuffle() | broad.core.sequence.Sequence |  |
| unloadAllSequences() | broad.core.sequence.Sequence |  |
| unloadEncodedSequence() | broad.core.sequence.Sequence |  |
| unloadSequence() | broad.core.sequence.Sequence |  |
| uppercase() | broad.core.sequence.Sequence |  |


---

Generated on Fri Aug 15 2014 15:10:56 for ESAT by  

 1.8.7
